# Telemedicine, Isolation, and Anxiety: The Impact of COVID-19 Lockdowns on an 87-Year-Old Woman

**DOI:** 10.7759/cureus.36195

**Published:** 2023-03-15

**Authors:** Alfred Amendolara, Erika Noonan

**Affiliations:** 1 Department of Clinical Science, Noorda College of Osteopathic Medicine, Provo, USA; 2 Federated Department of Biology, New Jersey Institute of Technology, Newark, USA

**Keywords:** coronavirus disease 2019, isolation, geriatrics, mental health, telemedicine, anxiety, covid-19

## Abstract

This report describes an 87-year-old female who received cognitive behavioral therapy and medication for anxiety before, during, and after the coronavirus disease 2019 (COVID-19) lockdowns. Our objective is to highlight the impact of isolation, examine the use of telemedicine during the pandemic, and stress the importance of early implementation of this technology. To this end, a chart review of psychotherapy and psychiatry progress notes from 2019 to 2022 and a patient interview were utilized to assess the impact of COVID-19 and telemedicine on the patient’s anxiety symptoms, feelings of isolation, and treatment plan. Feelings of isolation, especially, were exacerbated. Prior to the pandemic, the patient was extremely physically and socially active. The reduced ability to interact with others and maintain her independence was detrimental. As a result, COVID-19 impacted the patient’s progress significantly and caused regression of symptoms. However, telemedicine allowed for the continuation of therapy and follow-up to the present time. Though telemedicine allowed the patient to receive regular care for the duration of the lockdown and to regain control of anxiety symptoms, she only recently became comfortable with the technology. Now, the patient prefers the convenience and ease of telemedicine, continues to receive care through this modality, and feels that her current care is equivalent to in-person therapy. This case report should serve as a reminder of the effect that isolation can have on older adults with pre-existing anxiety. Notably, isolation may be related to the recent COVID-19 pandemic or other factors, such as reduced mobility or limited access to social services. In any case, isolation has a substantial impact on older patients’ mental health. And, despite the availability of telemedicine, clinicians should be aware of the technical challenges surrounding emergency implementation. We suggest early exposure to telemedicine for patients, as well as staff training focused on the potential technical limitations of those patients. We also suggest an assessment of technical literacy, conducted early on as part of a patient's initial intake. The main limitation of this report, and the conclusions drawn herein, is the lack of quantitative measures available. Thus, assessment of the patient’s condition and symptoms was restricted to clinician assessment and self-reported measures. We feel though that this remains a useful example of the long-term benefit of telemedicine for older individuals.

## Introduction

As of January 2023, coronavirus disease 2019 (COVID-19) has resulted in over one million deaths in the United States [1]. Older Americans have been disproportionately affected by this disease. According to the data available from the CDC, approximately 75% of those who died of COVID-19 were 65 years or older [2]. In addition to the physical toll COVID-19 has taken on older populations, there has been an impact on mental health [3]. Older patients have shown an increased incidence of depression and anxiety related to the pandemic [4]. For example, a 2021 study by Zaninotto et al. reports an increase in the prevalence of depressive symptoms from 12.5% to 28.5% [5]. These changes were likely connected to the increased social isolation that occurred as a result of pandemic lockdowns and mandatory social distancing [6-8]. Decreased availability of and access to services may have contributed substantially [9,10]. Additionally, media exposure to the rapidly rising toll of COVID-19, in combination with misinformation about vaccination safety and efficacy, may have influenced feelings of anxiety surrounding the pandemic [11,12].

COVID-19 lockdowns, in addition to impacting patients directly, impacted access to health services. Despite this difficulty, telemedicine emerged as an effective tool to deliver care [13]. However, obstacles to its widespread use became apparent. Regulatory and reimbursement restrictions presented challenges to implementation [14]. The initial investment in software and training may also have prevented smaller organizations from rapidly adopting this relatively new method of care delivery [15]. Additionally, even with efficient appropriate training and robust supporting technology, patients and providers were slow to accept the change in dynamics inherent to video encounters [16]. Delivery of mental healthcare via telemedicine did, and still does, have limitations, especially when considering the availability of technology and digital literacy.

It is clear that the pandemic impacted older patients disproportionately. Loneliness, especially, was highly correlated with an increased risk of non-accidental injuries, depressed mood, psychotic symptoms, relationship problems, and antidepressant use [5]. We feel it is important to document this case, which describes an increase in anxiety symptoms in an older American adult experiencing significant loneliness as a result of COVID-19 lockdowns. This case provides a clear example of the impact of isolation on an older adult and highlights the need for increased social services, the importance of activity in older adults, and the usefulness of telemedicine. We hope to show that despite the technical issues encountered by this patient, telemedicine remains an important tool for increasing access to care, especially in emergency scenarios, such as the COVID-19 pandemic.

## Case presentation

A chart review of therapy notes and psychiatry progress notes was conducted after obtaining patient consent. Notes were available from January 2019 until the present day. Reviewed notes included all reports from the initiation of treatment in 2019 to October 2022. In addition to a chart review, the patient was interviewed in person in January 2023. The interview included questions about the patient’s feelings on treatment, COVID-19, levels of anxiety, and feelings of isolation.

Initial presentation

An 83-year-old female presented for outpatient psychiatric evaluation and therapy in January 2019. She initially complained of general feelings of anxiety. Specifically, she described fearing loss of control, confusion when stressed or anxious, hypervigilance, irritability, loneliness, sleep disturbances, and episodes of shortness of breath and palpitations related to feelings of anxiety.

At the initial presentation, the patient did not have a previous psychiatric diagnosis or treatment. However, she reported a history of verbal abuse by her late husband and longstanding feelings of anxiety that worsened with age. Her anxiety symptoms were further exacerbated by her fear of abandonment. Additionally, she noted her mother had an unspecified history of mental illness. These symptoms impacted her daily life and relationships. She was in otherwise good health. Her only significant medical history was osteoarthritis in her knees, which did not significantly limit her mobility. She was able to perform all activities of daily living independently. The patient was very socially active prior to the pandemic. She lived in a suburban area outside of a medium-sized city, and thus had easy access to a variety of services, activities, and necessities. Her primary support system at that time consisted of friends she made at various clubs and organized seniors’ activities and her son, who lived nearby.

Treatment plan

The patient was prescribed escitalopram 10 mg daily and alprazolam 0.5 mg as needed. These medications remained constant throughout treatment. Additionally, she began therapy sessions every other week in January 2019 with a licensed clinical social worker (LCSW). This therapy consisted of cognitive behavioral therapy focused on changing the negative perception of events and relationships in the patient’s life as well as general supportive talk therapy. Specific techniques included teaching relaxation skills to deal with stressful situations, systematically challenging negative thought patterns, and providing general anxiety education to aid in patient understanding. These visits initially took place in person but transitioned to virtual visits in March 2020 due to the COVID-19 pandemic. The therapist and psychiatrist responsible for care did not change. At that time, therapy frequency increased to once per week. This treatment remains constant up to the writing of this case report.

Complicating factors and barriers to care

COVID-19 was the primary complication of this case as well as a major barrier to care. Feelings of isolation and anxiety increased significantly at the onset of the pandemic, based on self-reported symptoms as well as therapy and psychiatry progress notes. Symptoms, as measured by subjective patient reports and provider assessment, did not return to pre-pandemic levels until late 2022. Therapy notes indicate that the patient was concerned with COVID-19 infection, the health of friends and children, and the lack of social connection. Most prominently, the patient was unable to attend regular social functions for extended periods of time. It was also indicated that the patient felt stress and anxiety because of news reports detailing steadily increasing deaths, infections, and long-term disability related to COVID-19 infection. The patient was able to receive a COVID-19 vaccine once it became generally available. This reduced her feelings of anxiety related to COVID-19 infection but did not eliminate them.

Due to the pandemic, the office where the patient received therapy ceased seeing patients in person. This necessitated the use of telemedicine to continue therapy and medication management. The patient did not have access to a computer and was unable to use video conferencing software on her cell phone or iPad. Treatment was therefore delivered via voice call.

Outcome and follow-up

The patient's medications have not been modified at the time of writing and she continues to receive weekly therapy sessions via telemedicine. She has seen improvement in her symptoms of anxiety since initiating therapy and medication treatment. However, treatment goals changed significantly over the pandemic, shifting from the resolution of symptoms to maintenance. The patient showed significant improvement with the initiation of treatment prior to the COVID-19 pandemic. A notable change in the content of therapy sessions, as well as an increased frequency and intensity of symptoms, began in March 2020 (the beginning of the COVID-19 pandemic in the United States). Self-reported levels of anxiety and worry increased for the duration of the pandemic. Her participation in social activities, namely, organized events for elderly people, decreased substantially and remains decreased as of the latest follow-up, though she is now able to regularly see her friends, and has become more comfortable with using technology to communicate. The patient remains in otherwise good health and is able to complete activities of daily living independently. Although the patient has since returned to pre-pandemic levels of anxiety, her progress was stalled by the social isolation that resulted from lockdowns.

Case summary

The patient presented here displayed increased symptoms of anxiety and a self-reported increase in feelings of anxiety and worry. These increased symptoms coincided strongly with the initiation of COVID-19 lockdowns. She was otherwise healthy and developed no new medical problems over the course of treatment. This correlation is further strengthened by the patient’s self-reported feelings of isolation due to those lockdowns. She was unable to attend regular social events of older adults and was unable to see friends and family. Additionally, the patient values independence and her ability to care for herself. Fear of losing this ability increased anxiety during the long COVID-19 restrictions. Notably, the patient began experiencing increased anxiety about non-COVID-19-related health concerns during this period, seemingly triggered by her concern over COVID-19.

## Discussion

A recurring theme in the patient’s chart and interview was the reduction in socialization during and after the initial wave of the pandemic. Many of the social clubs and services the patient utilized were shut down. Some remained unopened three years later, at the time of this report. As a result, she was effectively isolated from outside contact for some time. Although the patient expressed that she was “able to handle it” at first, extended restrictions lead to increased experiences of anxiety and regression during therapy. This exacerbated her pre-existing fear of abandonment. It also resulted in a return to negative behaviors of comparison and jealousy, which the patient had learned to cope with in therapy prior to the pandemic. This experience was certainly not unique to the patient presented here. It has been well established that older adults are vulnerable to loneliness, loss of resources, and negative behavioral changes as a result of the COVID-19 pandemic [4-8].

Starting in March 2020, after having been seen in person since January 2019, the patient began receiving psychiatric care and therapy remotely. As evidenced by the gradual return to baseline following the initial waves of the pandemic, telemedicine provided an effective method of delivery. Notably, limitations in technical literacy prevented the patient from taking full advantage of telemedicine. She had an iPad, which she felt was her primary connection to the outside world. However, the patient was unable to use video calling and relied on audio-only calls. So, while she had access to technology, she was not able to use it effectively. This represented a significant limitation and was a potential weakness of her treatment.

During the interview, the patient stated that the pandemic significantly impacted her social life as well as her ability to leave the house. She expressed concern that she would not be able to return to her pre-pandemic level of activity. Her decreased social exposure and subsequently increased feelings of isolation were, again, not unique [6]. Nor was her lack of access to social services [10]. Of particular importance was the shift in therapy goals from improving self-awareness and coping skills to “survival” during the lockdown. The patient expressed that she felt that she would have been in a significantly worse state without telemedicine, as she was able to attend weekly sessions remotely. After some period of adjustment, the patient became quite happy with her regular telemedicine sessions. She enjoyed the convenience of avoiding travel, especially in inclement weather. However, she expressed that much of her comfort and acceptance of telemedicine stemmed from her familiarity with her therapist and psychiatrist. Had she not been well-established in the practice, it is not clear that her experience with telemedicine would have been positive.

Clinical implications

This case illustrates two important implications for clinicians working with older Americans. One, clinicians should be aware of technical and implementation issues surrounding telemedicine. Two, clinicians should be aware of the impact of isolation on older patients related to the COVID-19 pandemic and other causes such as reduced mobility, limited availability of social services, weak support system, or remote location.

Further expanding on the first clinical implication, clinicians must be aware of the technical challenges that may present when introducing telemedicine services. These challenges can arise in several areas. For instance, office policy can impact the delivery of telemedicine. Difficulty in scheduling and coordinating appointments is a concern, as well as potentially inadequate training of staff to navigate the issues that arise when older adults struggle with new technology. More fundamentally, issues with the technology itself, for example, unstable internet connections, increased bandwidth use due to video calling, or lack of appropriate devices in-office, can create difficulty in the delivery of quality care. Steps may be taken to mitigate these issues, though. Staff should be adequately trained in using telemedicine software and trained on how to handle common pitfalls. Digital literacy should be assessed early in all patients, and, if deficiencies are discovered, clear instructions and help should be provided. There currently exist a variety of methods for assessing digital literacy and this is a growing topic of research, especially in older adults [17]. Additionally, telemedicine should be introduced before it is critically needed, especially to those who may struggle with digital literacy. This would allow a smooth transition to telemedicine in a future crisis.

The second implication is more difficult to address. While many of the factors contributing to isolation are systemic in nature, for example, lack of social services or physical disability, until such time as they can be addressed at the community level, it is essential that clinicians be aware of the impacts of isolation in older adults in general as well as in conjunction with the COVID-19 pandemic. It is especially important to remain conscious of the ongoing effects of health anxiety and reduced socialization. Despite restrictions being lifted almost universally in the United States and elsewhere, older patients experienced a reduction in services and social activities available to them [7,10]. Patients with pre-existing anxiety or depression may be especially susceptible to these changes. Clinicians may not be able to directly influence any of these factors. They should, however, strive to identify possible sources of isolation, both obvious and obscure.

Limitations

This case report suffers from one major limitation. There is a lack of quantitative data available on this patient. Assessments of her progress were made by examining records of the patient’s self-reported symptoms, carefully synthesizing therapy notes, interviewing the patient, and discussing the case with the LCSW responsible for much of her care. However, no psychometric tests were administered to this patient. Thus, there is no standardized metric with which to measure progress. Despite this, we feel that this was sufficient evidence to support our conclusions.

## Conclusions

Overall, the patient was adversely impacted by the social isolation caused by the COVID-19 pandemic. Her inability to socialize or see family exacerbated underlying anxiety and relationship issues. Although telemedicine provided a means to continue care, it was ultimately limited by technology literacy, and, despite continued access to therapy, treatment goals changed significantly over the course of the pandemic. As the population of the United States ages, these topics become more and more relevant to clinicians, especially those dealing primarily with older demographics. Ongoing observation and research should be conducted in this area and clinicians should be aware of the implications to current practice.
